# An asymptomatic neoformation on the hard palate

**DOI:** 10.11604/pamj.2021.38.145.22719

**Published:** 2021-02-09

**Authors:** Valentino Natoli, Cinzia Casu

**Affiliations:** 1 Private Dental Practice, Fasano, Italy,; 2 Private Dental Practice, Cagliari, Italy

**Keywords:** Hard palate lesion, traumatic fibroma, pyogenic granuloma

## Image in medicine

A 70-year-old patient with positive anamnesis of hypertension and arthritis, went to our observation for an asymptomatic localized lesion on the hard palate. The lesion has been present for about seven years and initially appeared as a slight swelling at the midline of the hard palate. He reported a slow and constant expansion but remained asymptomatic. The objective examination revealed poor oral hygiene conditions, previous periodontal disease and prosthetic and endodontic treatments. On the anterior third of the hard palate in a central position there were multiple pink rounded neoformations, the bigger of about 1 cm in diameter and of elastic consistency. The patient confirmed the habit of tapping the tip of the pen on the palate. Palpation of the laterocervical lymph nodes was negative. There was no leakage of liquid to compression.

**Figure 1:**
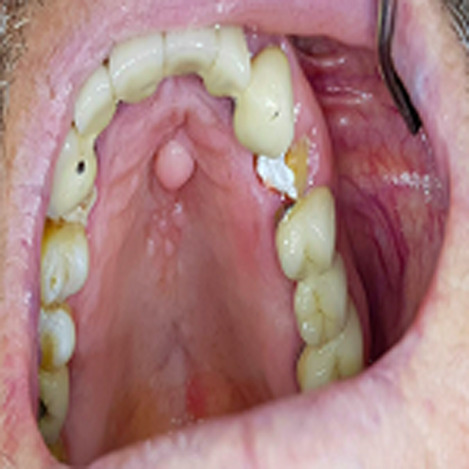
particular traumatic lesion on the hard palate

